# Intracellular Metabolite Pool Changes in Response to Nutrient Depletion Induced Metabolic Switching in *Streptomyces coelicolor*

**DOI:** 10.3390/metabo2010178

**Published:** 2012-02-17

**Authors:** Alexander Wentzel, Havard Sletta, Stream Consortium, Trond E. Ellingsen, Per Bruheim

**Affiliations:** 1 Department of Biotechnology, SINTEF Materials and Chemistry, Sem Sælandsvei 2a, N-7465 Trondheim, Norway; Emails: havard.sletta@sintef.no (H.S.); trond.e.ellingsen@sintef.no (T.E.E.); 2 Department of Biotechnology, Norwegian University of Science and Technology, Sem Sælandsvei 6/8, N-7491 Trondheim, Norway; Email: per.bruheim@biotech.ntnu.no (P.B.); 3 Coordinator: E. M. H. Wellington, Biological Sciences, University of Warwick, Coventry, CV4 7AL, UK; Email: e.m.h.wellington@warwick.ac.uk

**Keywords:** * Streptomyces coelicolor*, secondary metabolism, transition phase, metabolic switching metabolite profiling, LC-MS, GC-MS

## Abstract

A metabolite profiling study of the antibiotic producing bacterium *Streptomyces coelicolor* A3(2) has been performed. The aim of this study was to monitor intracellular metabolite pool changes occurring as strains of *S. coelicolor* react to nutrient depletion with metabolic re-modeling, so-called metabolic switching, and transition from growth to secondary metabolite production phase. Two different culture media were applied, providing depletion of the key nutrients phosphate and L-glutamate, respectively, as the triggers for metabolic switching. Targeted GC-MS and LC-MS methods were employed to quantify important primary metabolite groups like amino acids, organic acids, sugar phosphates and other phosphorylated metabolites, and nucleotides in time-course samples withdrawn from fully-controlled batch fermentations. A general decline, starting already in the early growth phase, was observed for nucleotide pools and phosphorylated metabolite pools for both the phosphate and glutamate limited cultures. The change in amino acid and organic acid pools were more scattered, especially in the phosphate limited situation while a general decrease in amino acid and non-amino organic acid pools was observed in the L-glutamate limited situation. A *phoP* deletion mutant showed basically the same metabolite pool changes as the wild-type strain M145 when cultivated on phosphate limited medium. This implies that the inactivation of the *phoP* gene has only little effect on the detected metabolite levels in the cell. The energy charge was found to be relatively constant during growth, transition and secondary metabolite production phase. The results of this study and the employed targeted metabolite profiling methodology are directly relevant for the evaluation of precursor metabolite and energy supply for both natural and heterologous production of secondary metabolites in *S. coelicolor*.

## 1. Introduction

The bacterial genus *Streptomyces* is well known for the production of numerous clinically used antibiotics. These filamentous soil bacteria undergo a complex developmental cycle, and antibiotic production usually occurs as part of the secondary metabolism of non-growing stationary cultures. The antibiotic biosynthetic pathway enzymes are induced while cell growth ceases during the transition from growth to secondary metabolism [1]. The molecular understanding of this cellular reorganization taking place during this transition phase is of importance for improving the strain's antibiotic producing capabilities. Several studies focusing on gene expression have been undertaken to reveal more about this complex metabolic switch [2,3]. However, analyses at the protein, metabolite and metabolic fluxes level are also needed to gain a more complete picture and understanding of cellular behavior and properties. In this regard, systems biology has emerged as a concept for integrating global experimental datasets covering several levels of metabolism using statistical tools and mathematical modeling [4].

*S. coelicolor* is the most-studied streptomycete, and sequencing of the genome revealed the presence of more than 20 cryptic gene clusters for secondary metabolites in addition to the well known secondary metabolites actinorhodin and undecylprodigiosin [5]. *S. coelicolor* has been intensively studied at the molecular and cellular level, and time-course gene expression studies have revealed that the metabolic switch consists of multiple finely orchestrated switching events already many hours before the classically defined transition phase [6,7]. Follow-up studies on knock-out mutants confirm this complex picture [8,9]. As this filamentous bacterium grows in heterogeneous pellets, it has been difficult to perform reproducible cultivations in shake flasks. Recently, we introduced a dedicated optimized batch fermentation strategy as part of a technical platform for generating reproducible expression data of *Streptomyces coelicolor* [10]. The central processes inside the cell during transition phase are turning off biosynthesis of cell building blocks while preserving synthesis of antibiotic precursor metabolites and energy production for maintenance purposes.

The comprehensive analysis of intra- and extracellular metabolite pools is called metabolomics, and mass spectrometry (MS) and nuclear magnetic resonance (NMR) have become the two most popular technologies in this field [11]. The advantages of MS are the high sensitivity and excellent resolution when combined with gas and/or liquid chromatographic (GC/LC) separation of complex biological extracts. GC/LC-MS analysis is either performed with a non-targeted or a targeted strategy. The former approach aims at identifying metabolites (e.g., biomarkers) of the biological system under study, while the latter aims at quantifying known compounds. Targeted analysis is of particular interest in systems biology since quantified metabolite pool data can be used directly in the integrated data analysis [12]. As the metabolome comprises compounds with a wide diversity in physico-chemical properties, ranging from highly charged to hydrophobic metabolite species, it is necessary to use several GC-MS and LC-MS methods for a comprehensive coverage of the most abundant metabolites [13,14]. In particular, LC-MS has become an invaluable tool in profiling families of secondary metabolites [15,16,17], and an LC-MS based method has only recently been applied in a metabolite analysis study of a synthetic metabolic switch in *S. coelicolor* [18].

In the present study, we have used both GC-MS and LC-MS/MS to monitor changes in the intracellular pools of important metabolite groups like amino and non-amino organic acids, phosphometabolites and nucleotides during the entire lifetime of *S. coelicolor* batch fermentations including in particular the period of metabolic switching to antibiotic production phase. The term 'metabolic switching' is nowadays often used in the sense of describing the molecular events during the transition from primary to secondary metabolism, and in the present study, we have chosen to use the term in this broader molecular sense. To our knowledge, this is the first report of a high resolution time-course metabolic profiling study in *Streptomyces* spp. to understand how the primary intracellular metabolite pools change in response to metabolic switching from growth to secondary metabolite production phase.

## 2. Results and Discussion

### 2.1. Batch Cultivations with Time Series Metabolite Sampling

**Figure 1:**
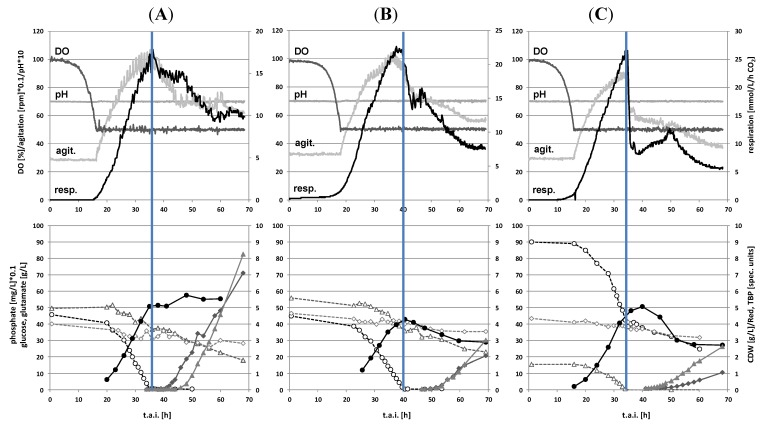
Batch cultivation on-line (upper panels) and off-line data (lower panels) obtained for (**A**) strain M145 on phosphate depletion medium SSBM-P; (**B**) strain INB201 (Δ*phoP*) on phosphate depletion medium SSBM-P; and (**C**) strain M145 on glutamate depletion medium SSBM-E. The time when the respective limiting nutrient is depleted from the medium is given as a vertical blue line. In the lower panels, dry weight concentrations are depicted as solid circles, glutamate concentrations as open triangles, glucose concentrations as open diamonds, phosphate concentrations as open circles, TBP (total blue pigments, *i.e.*, actinorhodins) as solid triangles, Red (undecylprodigiosin) as solid diamonds. The upper panels depict results from continuous monitoring of dissolved oxygen concentration (DO), pH, agitation (agit.) and CO_2_ concentration in the off-gas (resp.). t.a.i. time after inoculation.

In this study, we chose to include two cultivation conditions (phosphate and L-glutamate depletion during batch fermentation) and two strains (*S. coelicolor* A3(2) M145 wild type and the *phoP* deletion mutant INB201 [19,20] derived from M145, the latter only cultivated under phosphate limited conditions). The cultivation platform suitable for full-scale 'omics sampling [10], as well as the on- and off-line data for the two cultivations involving strain M145 discussed here have previously been reported elsewhere [6,7,8,9]. However, growth and secondary metabolite production profiles for the selected strains and cultivation conditions are repeated in this report as they are important for the interpretation of the metabolite profiling data. The medium needed supply of two carbon sources, D-glucose and L-glutamate, the latter also serving as the sole source of nitrogen in the medium, to establish a distinct transition between the growth and secondary metabolite production phases as well as to provide enough biomass for full-scale 'omics sampling already many hours prior to depletion of the respective limiting nutrient (phosphate in SSBM-P and L-glutamate in SSBM-E). The *S. coelicolor* M145 strain showed usual growth and secondary metabolite production onset profiles in the phosphate limited medium (Figure 1, left panel). After a period of linear growth, the culture experienced phosphate depletion 35 h after inoculation, preventing further growth and triggering the onset of secondary metabolite production. Undecylprodigiosin and actinorhodins were detected in the medium around 5 and 15 h after phosphate depletion, respectively, and in this intermittent period, the cells abandoned growth, induced the expression of secondary metabolite gene clusters and synthesized the corresponding proteins/enzymes. The *phoP* deletion mutant INB201 showed very similar growth and secondary metabolite production profiles as the M145 parental strain (Figure 1, middle panel), while the glutamate limitation in M145 wild-type clearly resulted in a much more dramatic physiological response, as seen in a rapid decline in CO_2_ evolution (Figure 1, right panel). The secondary metabolite production rates are significantly lower in the glutamate limited culture. However, the yield on carbon source basis is not particularly high (<2% w/w) for none of the two media applied (data not shown). In any case, the three cultivations presented here represent a good experimental design to explore how *S. coelicolor* cells adapt at the metabolite level to the changing cultivation conditions while reprogramming metabolism from growth to secondary metabolite production.

### 2.2. Metabolite Profiling of the Transition Phase

High resolution time-course metabolite profiling experimentation in *S. coelicolor* is challenging in many aspects. Firstly, the filamentous growth resulting in pellet morphology in submerged cultures pinpoints the need for carefully tested and well developed cultivation conditions to obtain reproducible data. Secondly, the optimized cultivation protocol uses two carbon sources, D-glucose and L-glutamate, and the latter compound complicates the MS analysis as a high extracellular concentration of L-glutamate makes it impossible to use differential sampling and extraction protocols [21]. Therefore, we employed a rapid filtration step with subsequent washing of the cell pellet in slightly hypertonic NaCl solution to remove the majority of extracellular metabolites. Thirdly, as some of the times-series included 36 sampling points, this challenged the strategy of the MS analyses. Samples to be compared (*i.e.*, within the same time-series, different cultivation conditions, different strains) therefore needed to be analyzed at different time points (days, weeks, months), and in between, maintenance operations (*i.e.*, cleaning of ions sources, cutting and replacing of GC columns, replacing LC columns and mobile phases, *etc.*) of the LC-MS and GC-MS instruments needed to be performed. Some samples also needed to be run on different instruments. Our strategy became to include an extensive set of standard mixtures to be run before, in between and after the actual samples in each series of analyses, and a set of internal standards to post-run normalize for changes in instrumental performance was added to each sample. In addition, the order of the time-series samples was randomized in the MS sequences. The fourth challenge lies in the presentation and interpretation of the extensive metabolite profiling datasets. For the complete understanding of cellular behavior, the metabolite profiling data need to be analyzed in an integrated way together with gene expression and proteome profiling data, this being one of the major aims of System Biology [22]. However, such an integrated analysis of data characterizing the metabolic switching in *S. coelicolor* including data from this study and corresponding transcriptome [6] and proteome analyses [9] lies beyond the aim of the present study.

**Figure 2:**
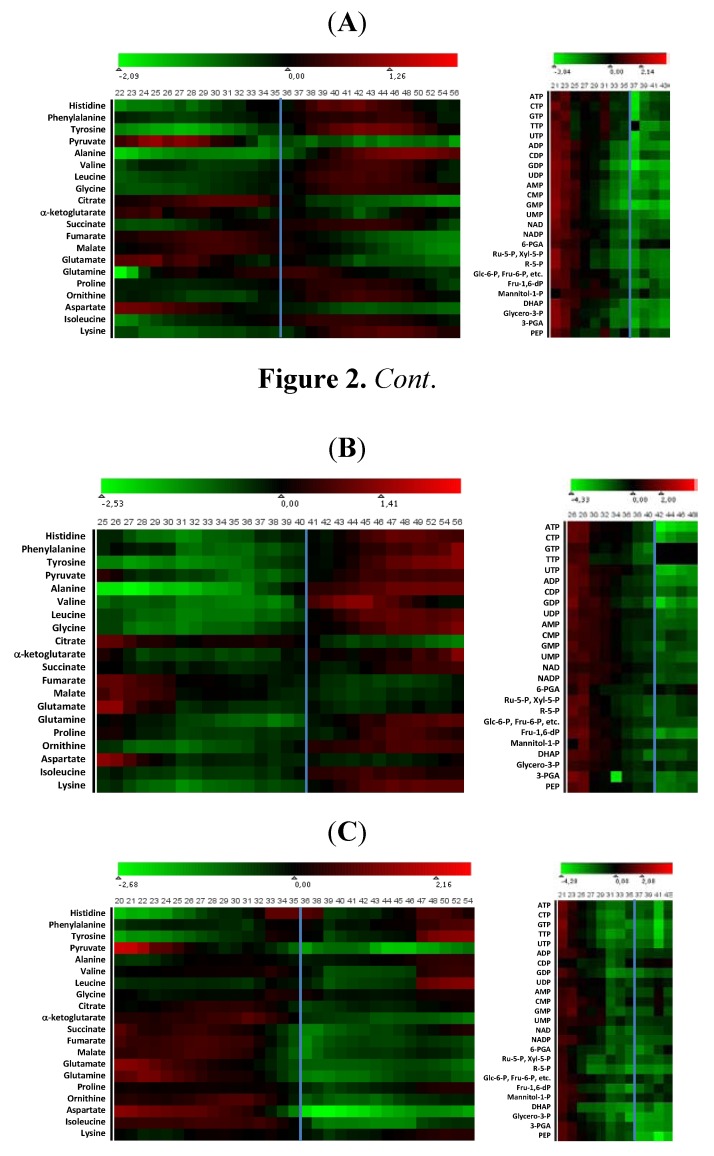
Time-course heat map representations of the 20 most abundant metabolites analyzed by the MCF GC-MS method (left hand side) and the 25 most abundant metabolites analyzed by the LC-MS/MS method (right hand side) detected in time-course samples of cultivations of (**A**) strain M145 on phosphate depletion medium SSBM-P; (**B**) strain INB201 (Δ*phoP*) on phosphate depletion medium SSBM-P; and (**C**) strain M145 on glutamate depletion medium SSBM-E. Metabolite pool data were CDW, log(2) and time-course average normalized; MCF GC-MS data were in addition normalized to the internal standard D_3_-alanine. GC-MS data were smoothed by applying a moving average over five adjacent time-course measurements (−2 to +2). Time-course data for each metabolite were weighted to the average of abundances of the entire series. Heat maps were generated using Mayday [33] with symmetrical scaling around zero as midpoint. The time when the respective limiting nutrient is depleted from the medium is given as a vertical blue line.

Extract analyses were performed in a randomized order. By that means, the time-course development of metabolite pools became more reliable to interpret as analytical biases were more evenly distributed over the time-course datasets. Figure 2 presents log(2) and series average normalized heat maps for the 20 and 25 most abundant metabolites analyzed with the GC-MS method and LC-MS/MS method, respectively. The whole nucleotide pool and the other phosphorylated metabolite levels analyzed with the LC-MS/MS declined in the M145 WT phosphate limited culture (Figure 2A, right panel). The decline started early in growth phase over ten hours before phosphate depletion in the medium. It is important to realize that streptomycete pellets are a heterogeneous cell mass where the innermost layers have lower metabolic activity or are even dead due to oxygen and nutrient limitation. Processes of programmed cell death have been reported and monitored in vegetative mycelium of *Streptomyces* sp. [23,24]. The portion of metabolic active cells in the total cell mass therefore decreases as pellet size increases during the lifetime of a batch culture, implying a significant influence on and a possible overestimation of observed decreasing trends in the heat maps since the metabolite pools are normalized to total cell mass. In addition, a positive correlation between specific growth rate and intracellular nucleotide phosphate pool concentrations or specific productivities bacterial species is well established for different bacterial species [25,26]. As a consequence, the observation that the nucleotide pools in the present study of *S. coelicolor* are highest in early growth phase is therefore not unexpected since in this metabolically most active phase, the specific growth rate is highest and the portion of dead mycelium is relatively low. Later in middle and late growth phase, the specific growth rate/specific oxygen consumption rate gradually declines [27], giving rise to the observed decrease in the total nucleotide phosphate pool. Similar profiles of decreasing nucleotide phosphate pools likely also demonstrating this combined effect of increasing portions of metabolically inactive cell mass and decreasing specific growth rates have previously been reported, e.g., for different *Streptomyces* spp. [28,29,30].

Despite the decreasing nucleotide pool, the energy charge (EC) in *S. fradiae* was found to be constant [29], pointing to a significant influence of the mycelial live/dead ratio on the measured concentrations of the total nucleotide pools while the ratio of ATP, ADP and AMP concentrations was maintained. Also in our study, the EC values were found to be relatively constant around 0.5–0.6 (Supplementary Figure 1). It is of general concern in metabolite analysis that determined EC values can be due to biases introduced during sample processing. Nevertheless, there are reports that the use of EC as a metabolic integrity characteristic is not unambiguous, and there are also reports that modify the general perception of the EC. Van der Werf and co-workers for example [31] reported EC values below 0.1 for glucose grown *Pseudomonas putida* cells while fructose grown cells showed an EC above 0.8. Barrette and co-workers [32] measured EC values below 0.2 during nutrient limitation but showed that cells easily recovered when exposed to more nutrient rich conditions.

The only metabolite pool that is increasing after phosphate depletion is that of 6-phosphogluconic acid (6-PGA). The increase might be the result of a continued high flux into the PPP needed during growth phase to supply NADPH and precursor metabolites, and an accumulation of NADPH due to the cellular consequences of phosphate depletion leading to feedback inhibition of 6-PGA dehydrogenase by NADPH.

The same general trends in the metabolite pools analyzed by the LC-MS/MS method was observed for the *phoP* mutant (Figure 2B, right panel). The mutant grew slightly slower, and the culture ran out of phosphate about five hours later than the wild-type M145 as indicated by the blue line in Figure 2B. The only metabolite pool that increased was again that of 6-PGA as discussed above, similarly to the WT culture. Also the wild-type M145 L-glutamate limited culture showed the same general declining metabolite profile, and there is no obvious major re-organization of these metabolite pools during the dramatic down-shift in respiration following the glutamate depletion (Figure 2C, right panel). Interestingly, there is, for yet unclear reasons, a temporal decrease in trinucleotide pools and a corresponding increase in mono- and di-nucleotide pools around the time secondary metabolites are detected in the culture (around 41 h after inoculation).

Since the GC-MS samples were analyzed at a higher frequency than the LC-MS/MS samples, a moving average of 5 adjacent measurements (−2 to +2) was used for easier visualization of the general trends in these datasets. While there was a general decline in the metabolite pools analyzed by the LC-MS/MS for the M145 wild-type strain cultured on phosphate limited medium SSBM-P, the picture is more scattered for the metabolites analyzed by the GC-MS method (Figure 2A, left panel). Of the metabolites with precursors in the glycolytic pathway and pentose phosphate pathway, only the pyruvate pool declined while the other metabolite pools increased after phosphate depletion occurred. Interestingly, there was only one metabolite, succinate, in the TCA cycle that increased while all other TCA metabolite pools seemed to be drained after the growth phase ended. This observation is consistent with findings from our recent proteomic profiling study which indicated persistent high protein levels of enzymes belonging to the top half of the TCA-cycle [9]. The measured L-glutamate concentrations must be evaluated with caution as the extracellular concentration is high on SSBM-P and on SSBM-E prior to L-glutamate depletion, and the intracellular measurements might be affected by extracellular L-glutamate contamination not completely washed away during the sample processing steps. However, a significant decrease was, irrespectively of this, observed. The aspartate pool was also declining while the rest of the amino acids with TCA-precursors exhibited an increased pool size after the growth phase had ended. Similar changes in metabolite pool sizes were, with only one exception, observed for the *phoP* mutant when cultivated under the same phosphate limited conditions (Figure 2B, left panel). The exception was pyruvate which increased as all other metabolites with glycolysis-precursors in the *phoP* mutant while it decreased in the wild-type strain M145. The reason for this is not obvious but likely to be a downstream effect of the *phoP* deletion.

The overall picture for the changes in GC-MS metabolite pool composition of the L-glutamate limited M145 wild-type cultivation (Figure 2C, left panel) is quite different from the phosphate limited cultures, contrary to the LC-MS/MS metabolites. A decrease in pool size is observed for all TCA-metabolites and metabolites synthesized from TCA precursors. The pyruvate profile is similar to the respective phosphate limited cultivation. Clearly, this pool is strictly dependent on the growth phase and not on which nutrient is becoming growth-limiting. The glycine pool remained almost constant while the histidine, phenylalanine, tyrosine, alanine, valine and leucine pools were, to varying extent, increased later in production phase after L-glutamate depletion.

The immediate response of the culture to L-glutamate depletion at the metabolite level is obviously due to the dual function of L-glutamate as carbon and nitrogen source. When glutamate becomes depleted, a major reorganization occurs as the *S. coelicolor* cells are not able to increase the glucose uptake rate to compensate for the glutamate depletion. However, as the cells also experience nitrogen limitation, growth stops and therefore synthesis of biomass precursors is shut down. Interestingly, the combined effects of changing to one carbon source and turning of growth is detected at the amino acid and organic acid levels while the pools of phosphorylated metabolites and nucleotides are more or less unchanged during this transition period (Figure 2C).

### 2.3. General Discussion

**Figure 3:**
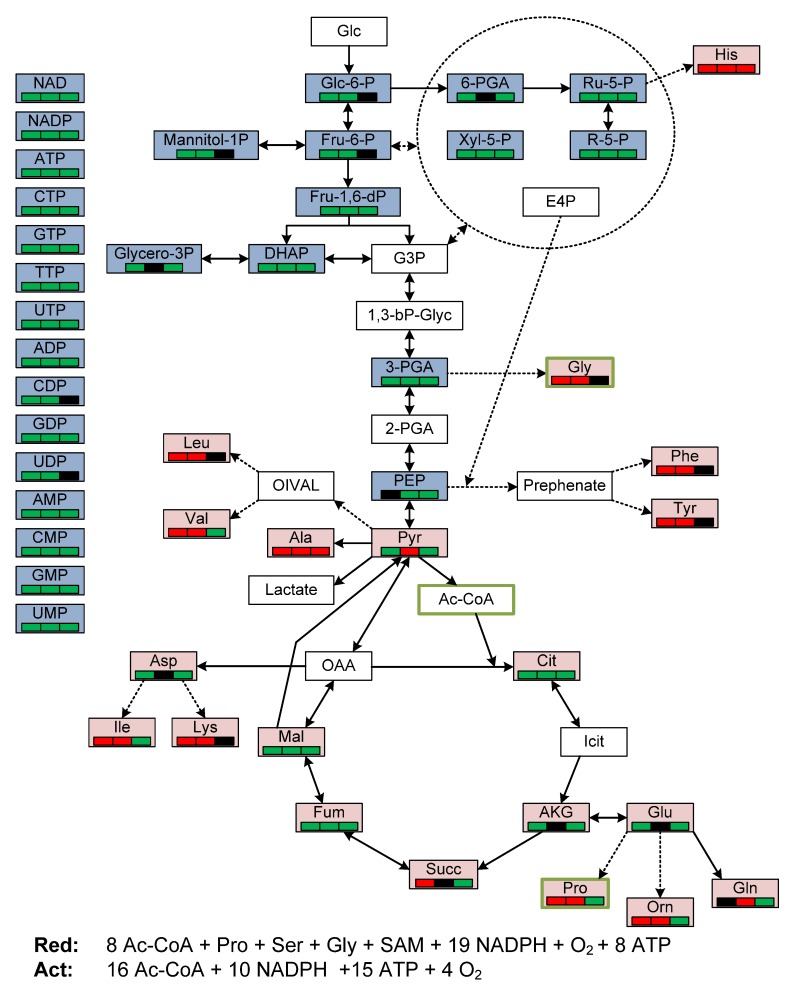
Scheme of central metabolic pathways in *Streptomyces coelicolor* with a special emphasis on metabolites covered by the present study (upper part). Blue color indicates metabolites detected by the LC-MS/MS method; red color indicates metabolites detected by the MCF GC-MS method. Lower part, stoichiometric equations for the biosynthesis of Act, Red and CDA. The precursor metabolites of Act and Red are indicated with green frame color in the metabolic network scheme. Colored boxes below metabolite names correspond to the three time-course experiments (left: M145, SSBM-P, middle: INB201, SSBM-P, right: M145, SSBM-E) and indicate the following metabolite profile classifications: 'red' (increased concentration in response to nutrient depletion), 'black' (no significant change in response to nutrient depletion), and 'green' (decreased concentration in response to nutrient depletion).

Figure 3 presents the core metabolism in *Streptomyces*; it might be that the Entner-Doudoroff pathway enzymes are also active as this has been shown for other Actinomycetes [34], and a recent proteome study of *S. coelicolor* M145 detected the ED enzyme 2-keto-3-deoxy-6-phosphogluconate aldolase [6,9]. The GC-MS method covers TCA metabolites and amino acids while the LC-MS/MS method covers the upper glycolytic pathway, pentose phosphate pathway metabolites and in addition the nucleotide pool, indicated with red and blue color in Figure 3, respectively. The overall trend in metabolite pool changes during the transition phase is also included in Figure 3 (*i.e.*, green bar for decrease, black bar for no change and red bar for increase) for the three cultivation situations (left bar for M145 in SSBM-P, middle bar for *phoP* deletion mutant INB201 in SSBM-P and right bar for M145 in SSBM-E). This simple visualization provides a quick overview of the results and shows a general decrease in intracellular nucleotide and phosphorylated metabolite pools, while there is both increased and decreased amino acid and organic acid pools for the two different growth situations and the two strains. Biosynthesis equations for Red and Act are also included in Figure 3, and their precursor metabolites are marked with green frames. The derivation of the stoichiometric equation for Red has not been published earlier but a thorough theoretical analysis of actinorhodin biosynthesis has been presented previously [35]. The important challenge for the cell during the transition phase is to maintain synthesis of precursor metabolites for secondary metabolite production, while the synthesis of biomass monomers is shut down. For actinorhodin synthesis, this implies that acetyl-CoA moieties need to be made available in addition to a significant amount of NADPH (produced either in the PPP or by isocitrate dehydrogenase in the TCA). Red synthesis is more complex as the amino acids proline, serine, glycine and the methyl-group donor *S*-adenosylmethionine (SAM) are required in addition to acetyl-CoA and a signification amount of NADPH. As long as the amino acid biosynthesis pathways are only feed-back inhibited at the protein level, the pools of these amino acids needed for Red synthesis should be maintained even in the absence of growth, but their synthesis might quickly become limiting in high productivity systems as exemplified by *Streptomyces lividans scbA* mutants overexpressing the pathway specific activator genes for Red and Act biosynthesis and obtaining yields over twenty per cent on carbon source basis [36].

That the physiological responses are different between a phosphate and a glutamate limitation can directly been seen on the CO_2_ respiration curves in Figure 1, *i.e.*, the sharp decrease in respiration in the glutamate limited culture. In this study, we can show that the difference in response to the different types of nutrient depletion is reflected also in the changes of the metabolite pool composition, and that this is mostly pronounced in the intracellular amino acid and organic acid pools. There is a general decline, starting already in early growth phase, in the pool levels of almost all metabolites in the glycolytic pathway and pentose phosphate pathway for both the phosphate and L-glutamate limited cultures. Expression of phosphate regulatory genes and genes for phosphate transport are up-regulated in the M145 wild type after phosphate depletion [6,9]. However, a direct effect of these events is not monitored at the metabolite pool levels, also supported by the high similarity at the metabolite level of the M145 wild type and the *phoP* deletion mutant. The observed nucleotide pool reductions in all three experiments can be explained by a combination of the relative decrease in amounts of metabolically active biomass where nucleotide phosphate pools are likely degraded to products in the dead mycelial fractions non-detectable by the two applied metabolite detection methods, and in general reduced nucleotide phosphate pools as a consequence of a reduced/zero specific growth rate.

The major concern at the metabolite level during secondary metabolism is sufficient supply of precursor metabolites and energy. Especially many secondary metabolites have a high demand of NADPH. This is usually solved at the cellular level by increasing pentose phosphate fluxes [37,38]. However, though these high demands on the molecule basis in principle also exist for Act and Red production (see formula in Figure 3 bottom), the relatively low production of these secondary metabolites are in the present case unlikely to be limited by NADPH pool levels.

## 3. Experimental

### 3.1. Strain and General Cultivation Parameters

Experiments were performed using *S. coelicolor* M145 and *phoP* deletion mutant INB201 derived thereof [19,20]. Spores prepared on SFM solid medium were used as the inoculum in all cultivations. Spores were germinated for 5 h at 30 °C and 250 rpm in 250 mL baffled shake-flasks containing 50 mL 2× YT medium and 2 g of 3 mm glass beads. Cultivations were performed in 3-liter fermentors (Applikon) with an initial culture volume of 1.8 L. The optimized growth medium used for studying the effect of phosphate depletion during batch fermentation (SSBM-P) consisted of Na-glutamate, 55.2 g/L; D-glucose, 40 g/L; MgSO_4_, 2.0 mM; phosphate, 4.6 mM; supplemented minimal medium trace element solution SMM-TE [39], 8 mL/L and TMS1, 5.6 mL/L. TMS1 consisted of FeSO_4_ × 7 H_2_O, 5 g/L; CuSO_4_ × 5 H_2_O, 390 mg/L; ZnSO_4_ × 7 H_2_O, 440 mg/L; MnSO_4_ × H_2_O, 150 mg/L; Na_2_MoO_4_ × 2 H_2_O, 10 mg/L; CoCl_2_ × 6 H_2_O, 20 mg/L, and HCl, 50 ml/L. The optimized medium for studying the effect of L-glutamate depletion (SSBM-E) was identical to SSBM-P except for the concentrations of Na-glutamate and phosphate adjusted to be 15 g/L and 9.2 mM, respectively. Clerol FBA 622 fermentation defoamer (Diamond Shamrock Scandinavia) was added to the growth medium before inoculation. Dissolved oxygen levels were maintained at a minimum of 50% by automatic adjustment of the stirrer speed (minimal agitation 325 rpm). The aeration rate was constant 0.5 L sterile air/L culture/min. Dissolved oxygen, agitation speed and CO_2_ concentration in off-gas were measured and logged on-line, while samples for the determination of cell dry weight, levels of key growth medium components and production of secondary metabolites were taken throughout the fermentation trials. For details on off-line analyses, it is referred to Nieselt *et al.* [6].

### 3.2. Sampling and Quenching

Samples for metabolite profiling were withdrawn from the cultivations in 1–2 h time intervals in the following cultivation periods: M145 on SSBM-P, 33 samples, 22–56 h after inoculation; INB201 on SSBM-P, 32 samples, 25–56 h after inoculation; M145 on SSBM-E, 36 samples, 20–56 h after inoculation (for exact sampling times, see Supplementary tables S4–S6). At each sampling time-point, 5 mL culture sample was withdrawn from the fermentation vessel and immediately applied to a 0.8 µm Supor 800 filter (Pall) placed on a magnetic filtration funnel (Pall) attached to a vacuum filtration manifold. On the filter disc, the cells were subsequently washed twice with one volume 2.63% (w/v) NaCl solution each, and immediately after the second washing step, the filter was transferred to a 50 mL tube containing 25 mL 60% methanol solution pre-chilled on an ethanol bath at −23 °C. The whole procedure from sampling to quenching of metabolism was completed within 10–15 s. The samples of each sampling point were collected on the ethanol bath at −23 °C, subsequently snap-frozen in liquid nitrogen and stored at −80 °C until metabolite extraction.

### 3.3. Metabolite Extraction

Samples stored at −80 °C were completely thawed on an ethanol bath at −23 °C. An internal standard mix was added to each 25 mL sample (biomass from 5 mL sample on filter in 25 mL 60% methanol solution) yielding final concentrations of 3.34 mM D_3_-alanine, 312.5 µM D_4_-succinate, 1.67 µM D_8_-valine, 62.5 µM ^13^C_6_-glucose, 0.416 µM ^13^C_10_, ^15^N_5_-adenosine monophosphate and 1.04 µM ^13^C_1_-α-ketoisocaproic acid). This standard mix included compounds to be used as internal standards in different analytical methods for metabolites with different chemical properties (organic acids, phosphometabolites, sugars). Samples were then subjected to three cycles of freezing on liquid nitrogen and thawing at −23 °C on the ethanol bath, found to be sufficient for reaching a maximum of compound extraction into the 60% methanol, and thereafter centrifuged for 5 min at −9 °C and 6000 × g. Supernatants were transferred to new tubes pre-chilled at −23 °C and then divided into aliquots á 6 mL in 15 mL screw cap tubes for analysis using different metabolite profiling methods. Samples were frozen at −80 °C and subsequently subjected to solvent evaporation on a freeze-dryer for 24 h. The freeze-dried samples were stored at −80 °C until MS analysis.

### 3.4. Metabolite Derivatization with Methyl Chloroformate (MCF) and GC-MS Analysis

Dried extract samples were dissolved in a solvent mixture consisting of 380 µL 1 M NaOH, 333 µL 100% MeOH, and 67 µL pyridine following a modified protocol of Villas-Boas *et al.* [40]. 20 µL 1 mM D_5_-glutamate was added to each sample as an analytical internal standard, and the dissolved sample was then transferred to a silanized 5 mL glass tube. While vortex mixing, the following steps were performed for derivatization with MCF, extraction with chloroform, and stopping the reaction with sodium hydrogencarbonate: 40 µL MCF added, 30 s vortex mixing, 40 µL MCF added, 30 s vortex mixing, 400 µL chloroform added, 10 s vortex mixing, 400 µL 50 mM NaHCO_3_ added, 10 s vortex mixing. The chloroform phase was dried with anhydrous Na_2_SO_4_ prior to GC-MS analysis. In general, 12 to a maximum of 24 samples were derivatized and subsequently analyzed in one sequence. In addition to the samples to be analyzed, each sequence contained several control runs including blank, chloroform and derivatized amino acid and organic acid standard mix samples before and after the biological samples to detect and potentially correct for instrumental variation during the sample series. GC-MS was performed using an Agilent 7890GC-5975MS system, EI source operated at 70 eV, equipped with a 30 m × 250 µm × 0.25 µm Agilent 122-5532G DB-5MS+DG capillary column. The data acquisition method was run in constant pressure mode with an operating pressure of 1 bar. D_5_-glutamate was used for retention time locking, which enabled retention time correction as columns were cut during maintenance operations. 2 µL sample was injected in pulsed split-less mode, and the metabolites were separated by using a 10 °C/min temperature gradient from 45 °C to 300 °C. The MS was operated in scan mode (start after 6 min, mass range 50–550 a.m.u. at 2.5 scans/s). The GC-MS data were analyzed semi-automatically using the Agilent ChemStation DRS (Deconvolution Reporting Software) and the AMDIS (NIST) deconvolution software using an in-house DRS library containing fifty metabolites. After automatic peak identification and integration, all compound peaks were inspected visually for the correct peak selection (retention time, qualifier ions) and the consistent peak integration, and manual correction was performed if necessary. To further assess the resulting dataset, the average, standard deviation, minima and maxima in retention time for respective compound peaks found in the 32 to 36 GC-MS runs (one time-series distributed over up to three sequences) were calculated. By that means, potential errors concerning peak choice were identified and corrected.

### 3.5. LC-MS/MS Analysis

LC-MS/MS analysis was based on the method introduced by Luo and co-workers [41] and performed on an Agilent 1200 series LC connected *via* an electrospray ion source to an Agilent 6410 triple quadrupole MS instrument. Forty-two common phosphorous containing metabolites were included in this MS/MS method, and collision energies were optimized for each individual metabolite. For the LC-MS/MS analysis, sequence variability was evaluated by quantification of the internal standards added to the samples prior to metabolite extraction.

### 3.6. Data Processing and Visualization

LC-MS/MS determined absolute metabolite concentrations in extracts were normalized to the CDW to give concentrations in µmol/g CDW. The processed LC-MS/MS data are given in Supplementary Tables 1–3. GC-MS data (relative abundances), quantified by peak integration (Agilent ChemStation DRS, AMDIS), were normalized to the cell dry weight (CDW) to give abundance per gram CDW and to the internal standard D_3_-alanine (divided by the D_3_-alanine abundance, multiplied with the average of all D_3_-alanine abundances of the respective entire time-series of samples). The processed GC-MS data are given in Supplementary Tables 4–6. For better visualization of general trends in metabolite pool changes, high resolution normalized time-series GC-MS data were smoothened by applying a moving average over five adjacent time-course measurements (−2 to +2). Time-course data for each metabolite were then weighted to the average of abundances of the entire series, and log(2) values were generated to obtain fold-change datasets. Heat maps were generated using Mayday [33] with symmetrical scaling around zero as midpoint (Figure 2). Energy charges (EC) were calculated by EC = ([ATP] + 0.5 * [ADP]) / ([ATP] + [ADP] + [AMP]).

## 4. Conclusions

A high resolution time-course metabolite profiling study of the transition phase in *S. coelicolor* has been performed to monitor intracellular metabolite pool changes in the wild type and a *phoP* mutant strain in response to phosphate and L-glutamate depletion. Targeted GC-MS and LC-MS methods were employed to quantify amino acid, organic acid, sugar phosphate and other phosphorylated metabolites, as well as nucleotide phosphate pools in time-course samples withdrawn from fully-controlled batch fermentations. 

A general decline was observed for nucleotide pools and phosphorylated metabolite pools for both the phosphate and L-glutamate limited cultures, likely due to a combination of a continuous decrease in amounts of metabolically active biomass during cultivation and in general reduced nucleotide phosphate pools as a consequence of a reduced/zero specific growth rate. However, the energy charge was found to be relatively constant during all phases of cultivation. Changes in amino acid and organic acid pools were found to be more scattered in the phosphate limited situation while a general decrease was observed in the L-glutamate limited situation. Results for a *phoP* deletion mutant strain showed basically the same metabolite pool changes as the wild-type strain when cultivated on the phosphate limited medium implying only little effect of the *phoP* deletion on the intracellular metabolite levels. 

This study shows that quantitative metabolite profiling is a valuable tool to provide information about metabolite pool changes during growth and onset of secondary metabolism. Mass spectrometric metabolite profiling combined with metabolic flux analysis might enlighten the precursor supply potential of *S. coelicolor*, which is of relevance for the potential usage of *S. coelicolor* as a host for heterologous expression of secondary metabolite gene clusters [42].

## Supplementary Files

Supplementary File 1 PDF-Document (PDF, 103 KB)

## References

[1] Chater K.F. (2006). Streptomyces inside-out: A new perspective on the bacteria that provide us with antibiotics. Philos. Trans. R. Soc. B-Biol. Sci..

[2] Hesketh A., Bucca G., Laing E., Flett F., Hotchkiss G., Smith C.P., Chater K.F. (2007). New pleiotropic effects of eliminating a rare tRNA from *Streptomyces coelicolor*, revealed by combined proteomic and transcriptomic analysis of liquid cultures. BMC Genomics.

[3] Lian W., Jayapal K.P., Charaniya S., Mehra S., Glod F., Kyung Y.S., Sherman D.H., Hu W.S. (2008). Genome-wide transcriptome analysis reveals that a pleiotropic antibiotic regulator, AfsS, modulates nutritional stress response in *Streptomyces coelicolor* A3(2). BMC Genomics.

[4] Zhou Z., Gu J.Y., Du Y.L., Li Y.Q., Wang Y.F. (2011). The -omics era- toward a systems-level understanding of *Streptomyces*. Curr. Genomics.

[5] Bentley S.D., Chater K.F., Cerdeno-Tarraga A.M., Challis G.L., Thomson N.R., James K.D., Harris D.E., Quail M.A., Kieser H., Harper D. (2002). Complete genome sequence of the model actinomycete *Streptomyces coelicolor* A3(2). Nature.

[6] Nieselt K., Battke F., Herbig A., Bruheim P., Wentzel A., Jakobsen O.M., Sletta H., Alam M.T., Merlo M.E., Moore J. (2010). The dynamic architecture of the metabolic switch in *Streptomyces coelicolor*. BMC Genomics.

[7] Alam M.T., Merlo M.E., Hodgson D.A., Wellington E.M., Takano E., Breitling R. (2010). Metabolic modeling and analysis of the metabolic switch in *Streptomyces coelicolor*. BMC Genomics.

[8] Waldvogel E., Herbig A., Battke F., Amin R., Nentwich M., Nieselt K., Ellingsen T., Wentzel A., Hodgson D., Wohlleben W. (2011). The P_ii_ protein GlnK is a pleiotropic regulator for morphological differentiation and secondary metabolism in *Streptomyces coelicolor*. Appl. Microbiol. Biotechnol..

[9] Thomas L., Hodgson D.A., Wentzel A., Nieselt K., Ellingsen T.E., Moore J., Morrissey E.R., Legaie R., Wohlleben W., the STREAM Consortium (2011). Metabolic switches and adaptations deduced from the proteomes of *Streptomyces coelicolor* wild type and *phoP* mutant grown in batch culture. Mol. Cell. Proteomics.

[10] Battke F., Herbig A., Wentzel A., Jakobsen O.M., Bonin M., Hodgson D.A., Wohlleben W., Ellingsen T.E., Nieselt K. (2011). A technical platform for generating reproducible expression data from *Streptomyces coelicolor* batch cultivations. Adv. Exp. Med. Biol..

[11] Villas-Boas S.G., Bruheim P. (2007). The potential of metabolomics tools in bioremediation studies. OMICS.

[12] Nielsen J., Oliver S. (2005). The next wave in metabolome analysis. Trends Biotechnol..

[13] van der Werf M.J., Overkamp K.M., Muilwijk B., Coulier L., Hankemeier T. (2007). Microbial metabolomics: Toward a platform with full metabolome coverage. Anal. Biochem..

[14] Kvitvang H.F.N., Andreassen T., Adam T., Villas-Boas S.G., Bruheim P. (2011). Highly sensitive GC/MS/MS method for quantitation of amino and nonamino organic acids. Anal. Chem..

[15] Geng P., Meng X.S., Bai G., Luo G.A. (2008). Profiling of acarviostatin family secondary metabolites secreted by *Streptomyces coelicoflavus* ZG0656 using ultraperformance liquid chromatography coupled with electrospray ionization mass spectrometry. Anal. Chem..

[16] Brautaset T., Bruheim P., Sletta H., Hagen L., Ellingsen T.E., Strom A.R., Valla S., Zotchev S.B. (2002). Hexaene derivatives of nystatin produced as a result of an induced rearrangement within the *nysC* polyketide synthase gene in *S. noursei* ATCC 11455. Chem. Biol..

[17] Bruheim P., Borgos S.E.F., Tsan P., Sletta H., Ellingsen T.E., Lancelin J.M., Zotchev S.B. (2004). Chemical diversity of polyene macrolides produced by *Streptomyces noursei* ATCC 11455 and recombinant strain ERD44 with genetically altered polyketide synthase NysC. Antimicrob. Agents Chemother..

[18] Jankevics A., Merlo M.E., de Vries M., Vonk R.J., Takano E., Breitling R. (2011). Metabolomic analysis of a synthetic metabolic switch in *Streptomyces coelicolor* A3(2). Proteomics.

[19] Rodriguez-Garcia A., Barreiro C., Santos-Beneit F., Sola-Landa A., Martin J.F. (2007). Genome-wide transcriptomic and proteomic analysis of the primary response to phosphate limitation in *Streptomyces coelicolor* M145 and in a delta*phoP* mutant. Proteomics.

[20] Santos-Beneit F., Rodriguez-Garcia A., Sola-Landa A., Martin J.F. (2009). Cross-talk between two global regulators in streptomyces: PhoP and AfsR interact in the control of *afsS*, *pstS* and *phoRP* transcription. Mol. Microbiol..

[21] Winder C.L., Dunn W.B., Schuler S., Broadhurst D., Jarvis R., Stephens G.M., Goodacre R. (2008). Global metabolic profiling of *Escherichia coli* cultures: An evaluation of methods for quenching and extraction of intracellular metabolites. Analy. Chem..

[22] Kirschner M.W. (2005). The meaning of systems biology. Cell.

[23] Manteca A., Fernandez M., Sanchez J. (2006). Cytological and biochemical evidence for an early cell dismantling event in surface cultures of *Streptomyces antibioticus*. Res. Microbiol..

[24] Yague P., Manteca A., Simon A., Diaz-Garcia M.E., Sanchez J. (2010). New method for monitoring programmed cell death and differentiation in submerged *Streptomyces* cultures. Appl. Environ. Microbiol..

[25] Dolezal J., Kapralek F. (1976). Physiological characteristics of chemostatically grown *Citrobacter freundii* as a function of the specific growth rate and type of nutrient limitation. Folia Microbiol. (Praha).

[26] O'Sullivan E., Condon S. (1999). Relationship between acid tolerance, cytoplasmic pH, and ATP and H+-ATPase levels in chemostat cultures of *Lactococcus lactis*. Appl. Environ. Microbiol..

[27] Rane K.D., Sims K.A. (1994). Oxygen uptake and citric acid production by *Candida lipolytica* Y 1095. Biotechnol. Bioeng..

[28] Liras P., Villanueva J.R., Martin J.F. (1977). Sequential expression of macromolecule biosynthesis and candicidin formation in *Streptomyces griseus*. J. Gen. Microbiol..

[29] Vu-Trong K., Bhuwapathanapun S., Gray P.P. (1980). Metabolic regulation in tylosin-producing *Streptomyces fradiae*: Regulatory role of adenylate nucleotide pool and enzymes involved in biosynthesis of tylonolide precursors. Antimicrob. Agents Chemother..

[30] Bascaran V., Sanchez L., Hardisson C., Brana A.F. (1991). Stringent response and initiation of secondary metabolism in *Streptomyces clavuligerus*. J. Gen. Microbiol..

[31] van der Werf M.J., Overkamp K.M., Muilwijk B., Koek M.M., van der Werff-van der Vat B.J., Jellema R.H., Coulier L., Hankemeier T. (2008). Comprehensive analysis of the metabolome of *Pseudomonas putida* S12 grown on different carbon sources. Mol. Biosyst..

[32] Barrette W.C., Hannum D.M., Wheeler W.D., Hurst J.K. (1988). Viability and metabolic capability are maintained by *Escherichia coli*, *Pseudomonas aeruginosa*, and *Streptococcus lactis* at very low adenylate energy charge. J. Bacteriol..

[33] Battke F., Symons S., Nieselt K. (2010). Mayday—Integrative analytics for expression data. BMC Bioinformatics.

[34] Gunnarsson N., Bruheim P., Nielsen J. (2004). Glucose metabolism in the antibiotic producing actinomycete *Nonomuraea* sp ATCC 39727. Biotechnol. Bioeng..

[35] Bruheim P., Butler M., Ellingsen T.E. (2002). A theoretical analysis of the biosynthesis of actinorhodin in a hyper-producing *Streptomyces lividans* strain cultivated on various carbon sources. Appl. Microbiol. Biotechnol..

[36] Butler M.J., Takano E., Bruheim P., Jovetic S., Marinelli F., Bibb M.J. (2003). Deletion of *scbA* enhances antibiotic production in *Streptomyces lividans*. Appl. Microbiol. Biotechnol..

[37] Becker J., Zelder O., Hafner S., Schroder H., Wittmann C. (2011). From zero to hero—Design-based systems metabolic engineering of *Corynebacterium glutamicum* for L-lysine production. Metab. Eng..

[38] Ikeda M., Ohnishi J., Hayashi M., Mitsuhashi S. (2006). A genome-based approach to create a minimally mutated *Corynebacterium glutamicum* strain for efficient L-lysine production. J. Ind. Microbiol. Biotechnol..

[39] Claessen D., Rink R., de Jong W., Siebring J., de Vreugd P., Boersma F.G., Dijkhuizen L., Wosten H.A. (2003). A novel class of secreted hydrophobic proteins is involved in aerial hyphae formation in *Streptomyces coelicolor* by forming amyloid-like fibrils. Genes Dev..

[40] Villas-Boas S.G., Delicado D.G., Akesson M., Nielsen J. (2003). Simultaneous analysis of amino and nonamino organic acids as methyl chloroformate derivatives using gas chromatography-mass spectrometry. Anal. Biochem..

[41] Luo B., Groenke K., Takors R., Wandrey C., Oldiges M. (2007). Simultaneous determination of multiple intracellular metabolites in glycolysis, pentose phosphate pathway and tricarboxylic acid cycle by liquid chromatography-mass spectrometry. J. Chromatogr. A.

[42] Gomez-Escribano J.P., Bibb M.J. (2011). Engineering *Streptomyces coelicolor* for heterologous expression of secondary metabolite gene clusters. Microb. Biotechnol..

