# Supplementary material for: Intracellular Metabolite Pool Changes in Response to Nutrient Depletion Induced Metabolic Switching in Streptomyces coelicolor
Source: Metabolites. 2012 Feb 17;2(1):178–94. doi: 10.3390/metabo2010178 (PMC3901196; doi:10.3390/metabo2010178)
Supplement: Supplementary File 1 — PDF-Document (PDF, 103 KB) [file metabolites-02-00178-s001.pdf]

Supplementary Material

Supplementary Figure 1. Energy charge (EC) values determined for time-course samples from cultivations of strain M145 on phosphate depletion medium SSBM-P (blue diamonds) and glutamate depletion medium SSBM-E (red squares), and INB201 (Δ*phoP*) on medium phosphate depletion medium SSBM-P (green triangles). The energy charge was determined by EC = ([ATP] + 0.5*[ADP]) / ([ATP] + [ADP] + [AMP]). The time when the respective limiting nutrient is depleted from the medium is given as a respectively colored dotted vertical line.

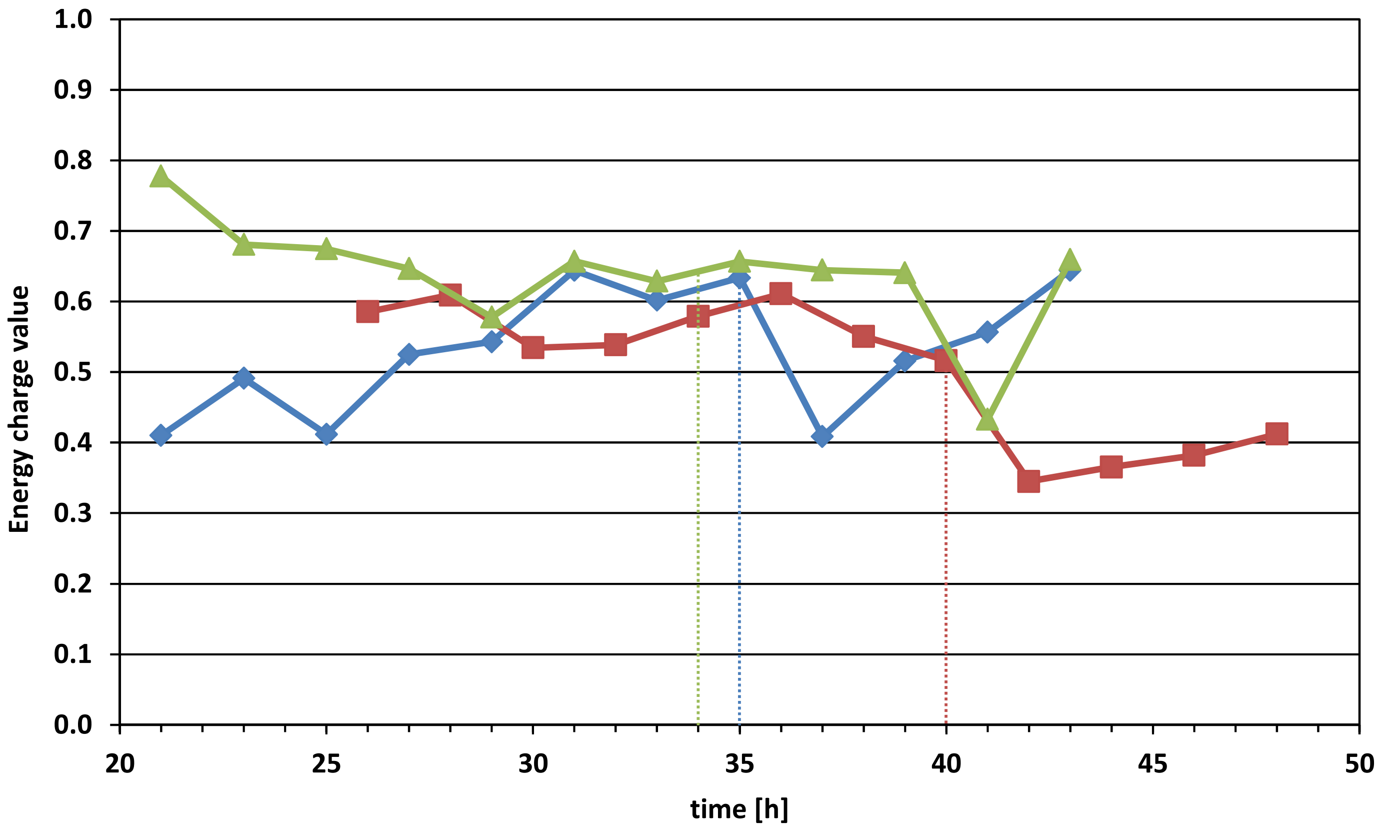

Supplementary Table 1. Original data of LC-MS/MS based phosphometabolite profiling of time-course samples from cultivation of strain M145 on medium SSBM-P. Absolute concentrations in samples [µM], normalized to CDW.

M145 - SSBM-P

**Sample No.**	**2**	**4**	**6**	**8**	**10**	**12**	**14**	**16**	**18**	**20**	**22**	**24**
**time [h]**	**21**	**23**	**25**	**27**	**29**	**31**	**33**	**35**	**37**	**39**	**41**	**43**
**CDW [g/L]**	**0.80**	**1.22**	**1.76**	**2.41**	**3.11**	**3.80**	**4.52**	**5.07**	**5.18**	**5.10**	**5.09**	**5.24**
**ATP**	1.29	1.84	0.72	0.98	0.85	1.18	0.52	0.49	0.07	0.20	0.31	0.36
**CTP**	0.19	0.12	0.05	0.07	0.05	0.09	0.04	0.04	0.01	0.02	0.03	0.04
**GTP**	0.24	0.30	0.08	0.12	0.11	0.20	0.08	0.08	0.01	0.05	0.05	0.07
**TTP**	0.05	0.03	0.01	0.02	0.02	0.03	0.01	0.01	*n.d.*	0.00	0.00	0.01
**UTP**	0.68	0.50	0.16	0.25	0.20	0.31	0.14	0.13	0.02	0.06	0.09	0.09
**ADP**	4.58	4.32	2.64	1.71	1.36	1.04	0.54	0.43	0.18	0.37	0.36	0.31
**CDP**	0.54	0.36	0.20	0.17	0.13	0.13	0.06	0.05	0.03	0.08	0.07	0.06
**GDP**	1.54	1.20	0.62	0.42	0.32	0.18	0.09	0.07	0.03	0.05	0.07	0.06
**UDP**	1.63	1.11	0.60	0.48	0.41	0.34	0.17	0.13	0.07	0.13	0.13	0.09
**AMP**	2.85	1.99	1.60	0.81	0.61	0.42	0.26	0.19	0.14	0.17	0.21	0.13
**CMP**	0.43	0.25	0.19	0.10	0.08	0.05	0.03	0.02	0.03	0.03	0.05	0.03
**GMP**	0.61	0.39	0.24	0.14	0.10	0.06	0.03	0.02	0.02	0.02	0.03	0.02
**UMP**	0.80	0.44	0.30	0.18	0.15	0.13	0.08	0.07	0.05	0.06	0.05	0.04
**NAD**	3.79	2.46	1.32	1.09	1.01	1.10	0.62	0.56	0.32	0.48	0.51	0.45
**NADP**	1.00	0.90	0.63	0.49	0.39	0.29	0.16	0.13	0.09	0.14	0.19	0.17
**6-PGA**	1.14	0.78	0.76	0.50	0.39	0.47	0.21	0.23	0.19	0.44	0.40	0.47
**Ru-5-P, Xyl-5-P**	0.81	0.38	0.34	0.20	0.11	0.12	0.06	0.06	0.04	0.07	0.06	0.06
**R-5-P**	0.71	0.35	0.27	0.16	0.09	0.10	0.06	0.05	0.03	0.06	0.05	0.04
**Glc-6-P, Fru-6-P, etc.**	14.28	10.62	6.07	5.32	5.37	5.48	2.83	2.77	1.74	2.99	1.65	1.43
**Fru-1,6-dP**	0.81	0.82	0.47	0.31	0.48	0.34	0.29	0.16	0.08	0.11	0.18	0.12
**Mannitol-1-P**	*n.d.*	0.06	0.06	0.05	0.05	0.05	0.02	0.02	0.02	0.03	0.02	0.02
**DHAP**	1.33	0.77	0.63	0.32	0.29	0.26	0.18	0.11	0.07	0.09	0.10	0.08
**Glycero-3-P**	2.69	1.67	0.81	0.51	0.48	0.36	0.20	0.20	0.14	0.21	0.17	0.13
**3-PGA**	7.39	5.04	2.30	1.65	1.01	1.42	0.57	0.65	0.28	0.55	0.41	0.32
**PEP**	0.26	0.19	0.09	0.07	0.07	0.18	0.06	0.10	0.03	0.07	0.04	0.05

Supplementary Table 2. Original data of LC-MS/MS based phosphometabolite profiling of time-course samples from cultivation of strain INB201 on medium SSBM-P. Absolute concentrations in samples [µM] , normalized to CDW.

INB201 - SSBM-P

**Sample No.**	**4**	**6**	**8**	**10**	**12**	**14**	**16**	**18**	**20**	**22**	**24**	**26**
**time [h]**	**26**	**28**	**30**	**32**	**34**	**36**	**38**	**40**	**42**	**44**	**46**	**48**
**CDW [g/L]**	**1.20**	**1.68**	**2.16**	**2.69**	**3.27**	**3.69**	**3.96**	**4.22**	**4.27**	**4.10**	**3.91**	**3.75**
**ATP**	2.80	2.64	1.24	1.00	1.01	0.85	0.52	0.40	0.04	0.06	0.08	0.10
**CTP**	0.28	0.24	0.10	0.09	0.09	0.08	0.05	0.04	0.01	0.01	0.02	0.02
**GTP**	0.22	0.22	0.11	0.09	0.11	0.08	0.04	0.03	*n.d.*	*n.d.*	*n.d.*	*n.d.*
**TTP**	0.06	0.07	0.02	0.02	0.02	0.02	0.01	0.01	*n.d.*	*n.d.*	*n.d.*	*n.d.*
**UTP**	0.68	0.52	0.32	0.28	0.28	0.24	0.16	0.14	0.03	0.02	0.04	0.04
**ADP**	3.59	3.20	1.91	1.58	1.10	0.77	0.69	0.57	0.21	0.22	0.26	0.23
**CDP**	0.43	0.31	0.22	0.17	0.13	0.10	0.09	0.08	0.04	0.04	0.07	0.05
**GDP**	1.13	0.54	0.59	0.41	0.21	0.15	0.16	0.10	0.02	0.04	0.07	0.05
**UDP**	0.79	0.55	0.57	0.50	0.37	0.27	0.26	0.24	0.10	0.08	0.11	0.10
**AMP**	1.46	1.13	0.96	0.75	0.58	0.40	0.36	0.35	0.18	0.19	0.21	0.19
**CMP**	0.13	0.11	0.11	0.09	0.06	0.04	0.04	0.04	0.02	0.04	0.03	0.02
**GMP**	0.19	0.13	0.12	0.10	0.07	0.05	0.04	0.04	0.02	0.03	0.03	0.03
**UMP**	0.24	0.27	0.18	0.14	0.13	0.09	0.07	0.08	0.05	0.05	0.04	0.03
**NAD**	2.45	2.54	2.40	2.38	1.99	1.63	1.31	1.23	0.66	0.74	0.81	0.79
**NADP**	0.47	0.58	0.42	0.35	0.26	0.21	0.18	0.13	0.09	0.12	0.11	0.10
**6-PGA**	0.68	1.12	0.46	0.36	0.36	0.33	0.30	0.40	0.43	0.33	0.42	0.59
**Ru-5-P, Xyl-5-P**	0.25	0.27	0.11	0.09	0.06	0.05	0.05	0.04	0.04	0.04	0.05	0.06
**R-5-P**	0.26	0.17	0.11	0.08	0.06	0.05	0.04	0.03	0.04	0.03	0.05	0.05
**Glc-6-P, Fru-6-P, etc.**	8.82	9.90	4.90	4.97	3.63	2.79	2.27	2.25	1.77	1.66	1.69	1.57
**Fru-1,6-dP**	1.28	1.45	0.68	0.50	0.40	0.27	0.18	0.15	0.07	0.10	0.08	0.09
**Mannitol-1-P**	*n.d.*	0.09	0.05	0.05	0.04	0.03	0.02	0.02	0.02	0.02	0.02	0.02
**DHAP**	0.66	0.62	0.43	0.35	0.23	0.16	0.13	0.06	0.10	0.10	0.09	0.14
**Glycero-3-P**	0.88	0.80	0.63	0.54	0.42	0.35	0.27	0.34	0.34	0.26	0.32	0.31
**3-PGA**	3.85	2.94	1.06	0.88	0.06	0.66	0.52	0.49	0.26	0.23	0.33	0.29
**PEP**	0.56	0.70	0.24	0.21	0.22	0.20	0.15	0.16	0.05	0.06	0.07	0.09

Supplementary Table 3. Original data of LC-MS/MS based phosphometabolite profiling of time-course samples from cultivation of strain M145 on medium SSBM-E. Absolute concentrations in samples [µM], normalized to CDW.

M145 - SSBM-E

Sample No.	4	6	8	10	12	14	16	18	20	22	24	26
time [h]	21	23	25	27	29	31	33	35	37	39	41	43
CDW [g/L]	0.81	1.25	1.73	2.26	2.95	3.72	4.33	4.69	4.91	5.04	5.05	4.92
ATP	13.83	7.49	4.30	2.90	1.54	1.09	1.12	1.85	1.32	1.45	0.47	1.62
CTP	1.81	0.87	0.51	0.37	0.16	0.11	0.13	0.31	0.19	0.26	0.07	0.29
GTP	4.22	1.40	1.13	0.68	0.35	0.25	0.28	0.21	0.20	0.24	0.08	0.25
TTP	0.64	0.29	0.16	0.12	0.06	0.03	0.04	0.07	0.04	0.05	0.02	0.06
UTP	4.14	1.82	1.50	1.04	0.56	0.38	0.48	0.45	0.45	0.50	0.18	0.53
ADP	7.31	6.95	4.69	3.78	3.01	1.18	1.55	2.03	1.75	2.08	1.97	2.10
CDP	0.35	0.32	0.21	0.23	0.22	0.09	0.13	0.29	0.24	0.38	0.33	0.38
GDP	0.90	0.61	0.31	0.25	0.21	0.07	0.08	0.10	0.09	0.13	0.16	0.14
UDP	1.23	1.17	1.01	0.86	0.89	0.34	0.50	0.39	0.54	0.64	0.78	0.66
AMP	1.34	1.67	0.86	0.73	0.72	0.29	0.35	0.48	0.34	0.36	0.92	0.33
CMP	0.30	0.34	0.24	0.18	0.16	0.05	0.07	0.13	0.10	0.10	0.18	0.09
GMP	0.17	0.26	0.11	0.08	0.12	0.03	0.03	0.04	0.03	0.04	0.10	0.04
UMP	0.31	0.33	0.27	0.28	0.28	0.10	0.16	0.11	0.10	0.12	0.13	0.12
NAD	4.78	3.57	3.50	2.79	2.00	0.91	1.30	0.90	1.30	1.40	1.52	1.84
NADP	1.63	1.49	0.88	0.64	0.49	0.26	0.27	0.27	0.20	0.26	0.27	0.28
6-PGA	2.08	1.15	0.89	0.81	0.73	0.38	0.56	0.37	0.25	0.39	0.71	1.17
Ru-5-P, Xyl-5-P	0.54	0.33	0.18	0.10	0.09	0.17	0.13	0.08	0.11	0.12	0.10	0.13
R-5-P	0.51	0.28	0.16	0.06	0.06	0.09	0.07	0.04	0.05	0.06	0.05	0.07
Glc-6-P, Fru-6-P, etc.	27.23	19.95	17.12	13.09	10.03	8.36	6.66	9.43	9.56	7.49	4.81	9.93
Fru-1,6-dP	1.74	1.46	0.50	0.42	0.39	0.18	0.21	0.12	0.22	0.35	0.56	0.46
Mannitol-1-P	0.20	0.14	0.12	0.08	0.07	0.03	0.04	0.05	0.05	0.06	0.04	0.07
DHAP	0.73	0.46	0.11	0.11	0.09	0.08	0.06	0.05	0.07	0.12	0.05	0.07
Glycero-3-P	5.41	3.15	2.60	1.78	1.47	0.69	1.13	0.38	0.42	0.53	0.54	0.67
3-PGA	8.94	6.05	3.08	2.59	2.11	1.11	1.66	1.52	0.51	0.62	0.63	0.72
PEP	2.85	1.90	1.29	0.98	0.71	0.43	0.56	0.42	0.14	0.15	0.06	0.12

Supplementary Table 4. Original data of MCF GC-MS based metabolite profiling of time-course samples from cultivation of strain M145 on medium SSBM-P. Relative abundances, normalized to CDW and internal standard D_3-alanine.

M145 - SSBM-P

**Sample no.**	**1**	**2**	**3**	**4**	**5**	**6**	**7**	**8**	**9**	**10**	**11**	**12**	**13**	**14**	**15**	**16**	**17**
**time [h]**	**20**	**21**	**22**	**23**	**24**	**25**	**26**	**27**	**28**	**29**	**30**	**31**	**32**	**33**	**34**	**35**	**36**
**CDW [g/L]**	**0.62**	**0.80**	**1.00**	**1.22**	**1.47**	**1.76**	**2.07**	**2.41**	**2.76**	**3.11**	**3.45**	**3.80**	**4.16**	**4.52**	**4.84**	**5.07**	**5.17**
**Histidine**	*n.d.*	11541	*n.d.*	11574	6969	16427	*n.d.*	12880	15459	15292	5989	22448	22640	29080	13080	28130	23087
**Glycine**	349651	383017	351402	399534	478022	269256	396210	374197	473261	446563	409826	512859	508598	526253	524095	553235	655936
**Tyrosine**	144340	134213	127664	113661	159978	90964	87982	86951	107908	112316	106675	132412	159562	176829	193226	226400	297421
**Phenylalanine**	95555	88903	99407	91949	110088	72542	72038	68515	93677	80929	82092	90306	115296	97723	100778	103001	143647
**Pyruvate**	71440	8273	90346	198036	56884	134056	169185	157487	44706	136893	95189	84873	16596	28131	65015	39331	6582
**Alanine**	4611238	4414454	4756083	4818384	5198859	6629272	8487094	7576446	7542380	7819097	7610658	7540644	7198439	7105279	7038519	6844037	8295247
**Leucine**	123595	107673	129239	116351	144970	153611	156339	146171	148940	131067	152548	169950	178914	172067	174002	173562	223787
**Valine**	1034525	1079923	1348753	1622179	1951698	2302601	2198553	1503407	1498922	1504333	1614381	1641096	1708974	1636192	1648050	1612386	2443857
**Citrate**	366788	420334	381372	463504	366316	404825	509112	547447	522115	545289	598583	690826	638264	464319	528940	567145	331683
**α-ketoglutarate**	76073	150389	66197	325651	51053	109964	119392	153185	68274	160610	127203	123585	51152	66288	110530	99094	53378
**Glutamate**	188638434	262253627	192510376	586923893	148859485	197585236	335080415	272648720	159215427	287923390	225889664	175449026	89605489	104001114	176389050	182203563	113010715
**Glutamine**	158308	148470	164105	161462	225116	545561	1593335	951159	574511	472617	681839	971022	909621	769439	931815	947710	947396
**Ornithine**	254108	306803	323496	383649	392310	347437	272530	260436	317351	293705	208610	283776	303690	293275	252337	350759	597637
**Proline**	1071054	1021730	1055301	1095604	1187536	985084	1074200	995399	1039732	1027333	1088869	1114232	1134214	1155192	1220107	1198091	1358790
**Succinate**	1186946	693495	1046390	902113	926146	913743	865494	924257	1044106	1339327	1346296	1353202	1360110	1636033	1733641	1582112	1471518
**Fumarate**	346025	235280	377941	383426	355693	353199	407086	371539	434968	452773	476811	438734	397337	341569	389399	341325	295259
**Malate**	199951	252749	266023	284109	294044	299378	309615	318145	369487	438452	425301	407037	323037	353940	356008	329111	275548
**Aspartate**	10749701	11186920	12362618	13439519	12796756	9195430	9915705	8243769	9156644	8234913	7107759	6525358	6425381	4913557	5463109	5502037	6271809
**Isoleucine**	205071	199238	186778	193987	198888	268700	406874	368224	334079	319691	353402	426498	417072	436657	420148	405950	560028
**Lysine**	663068	1554918	847759	1008223	754896	752667	652086	831969	914245	1032564	781751	1073474	951181	1036490	952591	1268271	1192560

**Sample no.**	**18**	**19**	**20**	**21**	**22**	**23**	**24**	**25**	**26**	**27**	**28**	**29**	**30**	**31**	**32**	**33**
**time [h]**	**37**	**38**	**39**	**40**	**41**	**42**	**43**	**44**	**46**	**48**	**50**	**52**	**54**	**56**	**58**	**60**
**CDW [g/L]**	**5.18**	**5.14**	**5.10**	**5.07**	**5.09**	**5.14**	**5.24**	**5.36**	**5.60**	**5.75**	**5.74**	**5.63**	**5.51**	**5.46**	**5.48**	**5.53**
**Histidine**	*n.d.*	20891	35853	50927	48539	27627	36216	48226	20540	33565	17698	18915	20904	19449	20313	23612
**Glycine**	421426	676084	817257	964897	910507	832926	792697	844060	823784	869193	729949	773331	710068	716766	590720	666308
**Tyrosine**	161565	319689	402960	633629	489866	497243	486296	650142	490863	548743	349329	329019	292177	263475	255432	341941
**Phenylalanine**	99554	135901	134362	188561	130810	134060	119310	180644	154914	199284	127944	108695	87883	77753	85036	126067
**Pyruvate**	*n.d.*	54663	49808	16977	34938	39466	34083	19621	35849	11667	51025	42318	28835	22613	22415	4323
**Alanine**	11834048	12776059	15608866	21053312	23529311	26493214	27546077	29554292	36243680	33050160	32765587	32127825	29740783	27170379	21143323	20109425
**Leucine**	202251	261921	276680	374696	302197	282760	255987	341744	284148	350607	201464	164983	166263	154801	181591	185159
**Valine**	2303621	2145910	2484236	3282394	3224689	3377851	3008716	3541302	3334365	3232962	2553159	2164129	2009044	1748945	1697489	1579013
**Citrate**	210927	281573	310335	242243	237411	217153	216526	213859	206712	214490	163883	169561	157737	137346	123697	177898
**α-ketoglutarate**	116145	121566	133485	42609	52508	76370	67536	56868	102865	83795	162238	172701	48860	51476	111260	81287
**Glutamate**	211628088	188269690	190337721	78590702	102243591	125575487	110337289	80796057	140575403	90879710	116002347	116716554	69384635	70280211	89507957	80331747
**Glutamine**	1045822	1148587	961577	841800	646383	789824	615528	693262	768608	655599	496686	552882	563593	557330	544950	679794
**Ornithine**	310964	383874	481873	676746	538861	516331	544358	651091	493809	555897	390725	423993	351325	369673	333091	363609
**Proline**	1358105	1504817	1556388	1975574	1599162	1481658	1410256	1716929	1475316	1658708	1234916	1042232	930121	789675	753836	806373
**Succinate**	1891128	1572508	1786090	1733424	1999021	1925388	1916990	1917781	1944628	1896695	1499510	1361711	1006115	818040	678908	814847
**Fumarate**	301707	293810	283177	260895	222624	203492	177853	209181	143380	173646	147586	159570	147113	128711	136193	137512
**Malate**	284686	293552	265802	263776	245287	218941	191104	200009	161036	151346	126352	140155	128652	110742	115176	115652
**Aspartate**	5410860	5184322	4502144	4919776	3572349	3493173	3424984	4195725	4077928	4648042	3872168	3988656	3444898	3398729	3290000	3527852
**Isoleucine**	512902	543299	536737	649320	571770	543592	502181	578740	528685	544935	442326	410221	412953	417109	329804	322851
**Lysine**	679414	1244132	1972166	2361937	1933087	1924705	2092146	2597663	2220625	2733899	1749688	1923086	1702671	1663640	1504995	1779418

Supplementary Table 5. Original data of MCF GC-MS based metabolite profiling of time-course samples from cultivation of strain INB201 on medium SSBM-P. Relative abundances, normalized to CDW and internal standard D_3-alanine.

INB201 - SSBM-P

**Sample no.**	**1**	**2**	**3**	**4**	**5**	**6**	**7**	**8**	**9**	**10**	**11**	**12**	**13**	**14**	**15**	**16**
**time [h]**	**23**	**24**	**25**	**26**	**27**	**28**	**29**	**30**	**31**	**32**	**33**	**34**	**35**	**36**	**37**	**38**
**CDW [g/L]**	**0.48**	**0.72**	**0.96**	**1.20**	**1.44**	**1.68**	**1.92**	**2.16**	**2.42**	**2.69**	**2.98**	**3.27**	**3.51**	**3.69**	**3.83**	**3.96**
**Histidine**	22043	38294	22737	12754	15735	23191	8960	7821	13796	12347	11328	12915	22675	14764	18695	14493
**Glycine**	803659	1398075	779415	445583	525509	846053	306524	473466	600092	635946	494799	568918	780168	650049	653918	675955
**Tyrosine**	249194	259944	107901	62364	110614	251095	110906	142875	147759	169481	123539	158427	194013	193265	195936	204291
**Phenylalanine**	383515	332863	220269	115214	231281	665076	171643	118002	198835	147504	156524	131389	217140	138774	173695	126097
**Pyruvate**	106611	115848	71324	27851	49437	52759	21353	41884	46675	37713	28466	28430	31721	22538	19969	21922
**Alanine**	9173016	10111487	5941843	3800147	4944383	9569136	5013866	7810860	11142620	9608789	8181853	9518843	16193255	13170338	13891137	17455933
**Leucine**	357467	420775	359704	192181	256914	379925	178037	189356	227788	212439	191680	198785	267510	217677	208365	189990
**Valine**	2979580	3099519	2798728	1901709	2890642	5395817	2001105	2731828	3145816	2401323	2087754	2286278	3102101	2417754	1978620	1867409
**Citrate**	1148051	1304390	1088626	530867	670012	1056120	381873	406368	617761	466397	469378	578022	683000	585154	525717	454615
**α-ketoglutarate**	263382	261830	218750	68721	91353	123710	90731	64181	125396	98304	75770	114082	91304	54125	133787	45830
**Glutamate**	703969826	1741127689	638886735	394138899	211111605	838138737	198008522	162004549	212709582	297464701	154376433	265405218	277881244	187693617	178432744	131908987
**Glutamine**	1090764	2283767	2575606	1656623	1416955	2344842	964758	1587286	1708151	978000	821294	824568	1398361	1056662	735780	574846
**Ornithine**	721174	970813	502110	368180	367510	641431	219154	318651	425897	417764	349958	417319	593880	529625	443269	517951
**Proline**	2083751	2672432	2169752	1302393	1544771	2138696	868466	1117896	1244019	1126241	947860	1081589	1368163	1084908	1024893	1006158
**Succinate**	1763916	1482367	1494063	1014893	745262	1774958	621087	1002755	1318192	1306074	784240	1396058	854265	939604	787721	977689
**Fumarate**	723575	794228	745236	534372	735277	828548	347215	382056	449398	383919	341909	371865	435694	309475	326772	242467
**Malate**	345018	610613	401459	461647	469609	868301	239034	335202	262424	260067	226941	273281	417613	310781	188896	208458
**Aspartate**	37647701	45151680	35392647	21540564	19287151	18814543	7142322	8453783	10695536	8906859	8111850	9253741	13123416	8962085	8310885	6804875
**Isoleucine**	514169	688308	691182	469754	565913	819993	276148	423614	531562	467067	432388	438980	711714	508580	417003	427377
**Lysine**	1716957	3030324	2111730	1665839	1229435	2351466	809218	943292	1162010	1118270	990237	1361281	1891250	1354764	1466043	1265388

**Sample no.**	**17**	**18**	**19**	**20**	**21**	**22**	**23**	**24**	**25**	**26**	**27**	**28**	**29**	**30**	**31**	**32**
**time [h]**	**39**	**40**	**41**	**42**	**43**	**44**	**45**	**46**	**47**	**48**	**50**	**52**	**54**	**56**	**58**	**60**
**CDW [g/L]**	**4.10**	**4.22**	**4.28**	**4.27**	**4.20**	**4.10**	**4.00**	**3.91**	**3.83**	**3.75**	**3.61**	**3.48**	**3.35**	**3.21**	**3.08**	**2.97**
**Histidine**	39974	17972	31896	14652	31134	27858	47650	54633	58027	56102	72620	29159	60463	32819	38997	63554
**Glycine**	1304356	896953	1463360	1046834	1914974	1015452	1282117	2351521	2437497	2369065	2789798	1155754	2789493	1866196	3662473	5085576
**Tyrosine**	390753	228866	386786	270893	538525	324405	452922	642738	772679	849887	942743	481734	1151086	763865	1074989	1434820
**Phenylalanine**	297919	158140	316216	237160	575608	210266	395023	372264	601768	630263	581794	263546	739447	455345	1005323	1731403
**Pyruvate**	49152	17216	59008	31216	103065	53134	73768	86268	103738	89422	82823	53227	108150	84990	98599	88274
**Alanine**	37762146	28313778	52169901	40581317	68829960	36757897	42918841	70847776	75703293	74355333	85324108	38340999	80707201	66405433	81677626	84632435
**Leucine**	381352	196141	413670	406955	1010709	622068	801647	1002774	1152398	920859	951641	443338	1085532	748674	1094990	1467244
**Valine**	3276530	1824648	4856362	10361691	26574400	12089917	11525592	12999137	10831305	8141355	7015474	2988796	5980498	4351697	5675513	6787484
**Citrate**	784645	422511	656573	219578	399434	189473	248044	408572	400496	308823	314495	128710	284044	183311	219367	215907
**α-ketoglutarate**	175183	85854	225634	100796	262437	87362	122758	104968	238449	251025	182377	64952	403635	85637	472908	765836
**Glutamate**	263288471	221752516	252984963	220345139	387759719	107481809	129852490	174450473	300215538	362451262	293337159	96896099	334580638	135286774	375813182	512747488
**Glutamine**	949411	610582	1489364	1476950	3603363	1415652	1733581	2318584	3606707	2874534	4679371	1876862	3276108	2995339	4666202	3667241
**Ornithine**	910439	511985	989195	837950	1898974	662887	860402	1060066	1442554	1512407	1736585	711589	1400590	1109006	1408086	1352337
**Proline**	1774473	1027794	1611832	917591	1674614	1305446	1994074	2892148	3570669	2914126	2937875	1327789	2677426	2002653	2093417	2044615
**Succinate**	1560563	1151211	1269475	1029753	1088414	886430	993289	1716477	1804976	1984880	2142821	1044644	2679731	1611533	2139990	2645130
**Fumarate**	489216	229882	371085	210938	385675	193793	203203	244829	268361	270382	273402	129176	333482	309262	393033	507988
**Malate**	291069	195957	248238	180997	284122	151231	156562	178794	251884	268595	278269	107346	258253	203614	322015	391707
**Aspartate**	13436226	7106011	12354405	7328967	15058680	6280179	7575278	9570698	11361264	9750485	11429939	6240159	14616695	12773909	15970587	18184456
**Isoleucine**	727909	460771	1008454	879718	1826850	727630	868757	1039263	1388940	1099056	1121465	457664	1019005	728907	1127947	1194726
**Lysine**	2532177	1353085	2047896	1715600	5049979	3126063	4236236	4588134	5715470	5478425	5703056	2748683	5892171	4632625	5529873	5804158

Supplementary Table 6. Original data of MCF GC-MS based metabolite profiling of time-course samples from cultivation of strain M145 on medium SSBM-E. Relative abundances, normalized to CDW and internal standard D_3-alanine.

M145 - SSBM-E

Sample no.	1	2	3	4	5	6	7	8	9	10	11	12	13	14	15	16	17	18
**time [h]**	**16**	**18**	**20**	**21**	**22**	**23**	**24**	**25**	**26**	**27**	**28**	**29**	**30**	**31**	**32**	**33**	**34**	**35**
**CDW [g/L]**	**0.20**	**0.37**	**0.63**	**0.81**	**1.02**	**1.25**	**1.49**	**1.73**	**1.98**	**2.26**	**2.58**	**2.95**	**3.34**	**3.72**	**4.06**	**4.33**	**4.53**	**4.69**
**Histidine**	*n.d.*	*n.d.*	6206	7170	3650	9467	5335	10443	8619	16903	15252	21393	21563	15627	15018	27757	46499	128905
**Glycine**	*n.d.*	956352	997437	587735	696002	768150	578408	746535	768829	734768	815532	746016	705639	767760	774309	826635	1263986	1618012
**Tyrosine**	50240	86718	70193	76679	78324	122422	74420	104243	108279	151354	175983	168565	174251	189267	186580	234842	247699	632662
**Phenylalanine**	209176	162266	219748	211036	220301	386045	196201	223963	295249	249313	330173	195956	207045	221999	253587	181588	513833	544609
**Pyruvate**	81447	188989	235837	50989	70652	62548	49853	36243	39667	18898	16945	21796	27541	21575	29650	35051	12878	8704
**Alanine**	6676224	6666149	5895201	4569064	5336345	4931403	6468917	8626743	8674003	8378227	8632713	8415645	8234258	8739511	9238084	9821564	5387746	7179304
**Leucine**	*n.d.*	210530	307568	256386	305775	338136	241762	277702	289289	301533	320481	270778	255715	269843	242705	258976	517418	573981
**Valine**	1178535	1727594	1981075	1617332	1934991	1656568	1861534	1891310	1830572	1919448	2122645	1790386	1889551	2112206	1962517	1929095	2170206	3431001
**Citrate**	*n.d.*	300264	641047	432245	433920	362573	499646	601640	664089	570962	621438	669760	607668	605139	644744	641688	341640	356456
**α-ketoglutarate**	79982	89277	141583	69847	66379	76988	100902	111095	105536	79865	62029	141503	145080	105341	111027	158106	71265	47893
**Glutamate**	214533249	306451143	394044891	292030748	212242485	177345984	182598435	178183663	159328516	116016078	127224915	119739515	112409930	106868266	102585783	100697788	56445651	51314935
**Glutamine**	*n.d.*	173402	1089222	1739307	1218705	1255651	1162141	954606	816233	632175	783113	630762	626607	685646	582593	710306	258906	189089
**Ornithine**	*n.d.*	319286	304604	271273	230376	361773	232986	278311	223591	320107	421778	387479	382793	398515	355529	453782	53824	142739
**Proline**	1090749	1212059	1547547	1186987	1335244	1328900	1080083	1284979	1321352	1305970	1361009	1218017	1203693	1274384	1232485	1308296	1225256	1389485
**Succinate**	1616226	1368444	1033221	675999	517980	694590	576444	998258	716665	946320	821714	673614	644938	607372	627492	644135	296315	171090
**Fumarate**	626558	418347	546111	384792	363233	298814	434211	496607	496766	466444	383598	470647	454987	362915	374613	394678	69287	96665
**Malate**	308414	299694	520279	452917	353243	321880	409869	482906	516844	435001	476653	405481	411466	354765	353929	311547	51572	90132
**Aspartate**	15308196	18764807	17193423	15993152	14497996	11149926	10556696	13937594	12874282	11551221	10675185	9290840	8595825	8084551	7436161	6986593	1866682	888753
**Isoleucine**	*n.d.*	280128	447457	441667	508829	412153	350101	438218	464104	460247	540823	421403	406128	444820	408359	409945	630711	166678
**Lysine**	*n.d.*	1520396	988704	1219549	1021009	835779	744551	1125926	1065145	1427362	1718838	1280110	1168101	1221139	1093800	1400952	1344774	1610668

Sample no.	19	21	22	23	24	25	26	27	28	29	30	31	32	33	34	35	36
**time [h]**	**36**	**38**	**39**	**40**	**41**	**42**	**43**	**44**	**45**	**46**	**47**	**48**	**50**	**52**	**54**	**56**	**58**
**CDW [g/L]**	**4.81**	**4.99**	**5.04**	**5.06**	**5.05**	**5.00**	**4.92**	**4.81**	**4.65**	**4.46**	**4.24**	**3.98**	**3.47**	**3.04**	**2.81**	**2.74**	**2.75**
**Histidine**	15711	15141	13347	23936	8659	17443	24487	17930	19292	28299	33387	34152	82481	21185	20720	9732	8423
**Glycine**	475616	640691	760222	795755	695518	713644	702404	684744	743431	724335	812428	804374	1563513	782271	856581	764984	769815
**Tyrosine**	89601	128093	142979	204476	149419	210637	222601	195564	225226	231002	242770	289721	1417104	810764	455524	353776	326178
**Phenylalanine**	182060	235913	300709	240891	207767	428736	247445	255139	460294	308898	413474	311392	812953	559384	629806	471246	389020
**Pyruvate**	3966	5268	12823	27897	15226	4925	4931	7615	7562	7342	5941	4255	15364	9229	14988	11213	11096
**Alanine**	6353763	6957837	7150268	6985442	6818396	7877045	7947142	8028158	9211739	8898009	9234377	10738465	15291120	10858869	11274747	10117580	9213116
**Leucine**	133913	187068	197218	257268	227995	238885	251182	231079	285148	274395	308995	329861	2561957	738573	717691	539657	443026
**Valine**	876182	989060	970931	1063888	918080	1116610	1084174	996495	1137353	1108291	1129904	1400937	6251561	2292691	1823711	1427903	1088064
**Citrate**	220168	196365	279894	458209	397809	335614	369527	335232	349963	357710	386344	467445	547078	361581	511682	615569	522184
**α-ketoglutarate**	37324	39598	52894	62120	43998	26507	37643	48018	32031	39672	35074	36617	47981	25825	26115	23614	18475
**Glutamate**	51536313	51970570	58285694	52068037	51338390	58111390	51211167	60231888	65162178	66885776	73783475	61148172	57797837	47835580	47096428	40082941	32466416
**Glutamine**	181515	201326	279074	191888	235468	299543	201368	302657	365429	360090	463261	317336	327292	275694	379557	167280	254617
**Ornithine**	153239	109057	98495	146496	49796	116478	121552	120308	93704	118943	227970	207686	136376	115815	208152	202785	238384
**Proline**	495884	623808	713975	783747	776748	905629	932655	911660	1103502	1054271	1067965	1168333	2459873	1434183	1345979	1176688	979729
**Succinate**	161434	205291	240413	295632	414347	236896	344824	291727	414943	375045	416776	552752	554848	577878	918123	712861	777399
**Fumarate**	108995	106808	114334	221076	201761	175598	143208	159509	179590	189454	188183	227803	242585	122281	147690	145558	124226
**Malate**	126189	117109	129533	223513	232462	173418	162568	172353	218617	212104	233682	246027	247766	191308	180020	119538	109029
**Aspartate**	964439	968544	1020523	900801	803318	1366576	1480063	1521646	1884628	1960940	2081130	2430344	2603267	1900283	1769020	1365541	1249236
**Isoleucine**	100860	113986	116759	138332	107147	115004	106764	96414	111037	105335	117332	122668	415114	189795	178005	154651	115260
**Lysine**	607908	911941	994261	913423	637607	1259228	1246941	1057873	1152688	1354501	1616409	1544286	1858303	1352822	1850357	1377850	1586759
